# Neutrophil-Lymphocyte Ratio and LDH/Albumin Ratio as Biomarkers for Severity and Mortality in Acute Pancreatitis

**DOI:** 10.5152/tjg.2025.24828

**Published:** 2025-06-16

**Authors:** Göksel Bengi, İbrahim Çelik, Süleyman Dolu, Soner Önem, Müjde Soytürk, Serkan Rendeci, Ömer Topalak, Hale Akpinar

**Affiliations:** 1 Department of Gastroenterology, Dokuz Eylül University Faculty of Medicine, İzmir, Türkiye; 2 Department of Internal Medicine, Dokuz Eylül University Faculty of Medicine, İzmir, Türkiye; 3 Department of Gastroenterology, Samsun Training and Research Hospital, Samsun, Türkiye

**Keywords:** Acute pancreatitis, LDH-Albumin Ratio, Neutrophil-Lymphocyte Ratio, prognosis

## Abstract

**Background/Aims:**

Acute pancreatitis (AP) is a common and potentially severe condition, and early identification of its severity is critical for appropriate clinical management. This study aimed to investigate the role of the Neutrophil-Lymphocyte Ratio (NLR) and Lactate dehydrogenase (LDH)/Albumin Ratio (LAR) in predicting the severity and prognosis of patients with AP and to determine the optimal NLR value.

**Materials and Methods:**

The demographic, clinical, and laboratory data of patients diagnosed with AP were retrospectively analyzed. Neutrophil-Lymphocyte Ratio was measured at admission (0 hours), and at 24 and 48 hours; C-reactive protein (CRP) values were recorded at 0 and 48 hours; and the LAR was calculated based on LDH and albumin values measured at 48 hours post admission. These values were compared with disease severity, mortality, organ failure, length of hospital stay, and the need for intensive care according to Ranson and bedside index of severity in AP (BISAP) scores.

**Results:**

According to the BISAP scoring, 38 patients (16%) were classified as having severe AP, while 200 patients (84%) had mild AP. The best parameter for predicting severe AP was found to be the 24-hour NLR with a sensitivity of 79% and specificity of 67%. The best parameter for predicting mortality and organ failure was the NLR at 48 hours. There was a statistically significant difference between the length of hospital stay and the need for intensive care with the CRP value at 48 hours. Additionally, there was a statistically significant relationship between LAR and mortality, length of hospital stay, organ failure, and the need for intensive care.

**Conclusion:**

This study demonstrates that the NLR and the LDH/Albumin Ratio are important and easily accessible markers for determining the severity and prognosis of AP. The NLR at 48 hours is an effective parameter for predicting mortality and organ failure, while the LDH/Albumin Ratio is significant in predicting mortality.

**Main Points**

- Neutrophil-Lymphocyte Ratio (NLR) and the Lactate dehydrogenase/Albumin Ratio are important, cost-effective, and easily accessible biomarkers for determining the severity and prognosis of acute pancreatitis (AP).
- The NLR at 48 hours was found to be a strong parameter for predicting mortality and organ failure.
- The best parameter for predicting severe AP was found to be the 24-hour NLR.
- There was a statistically significant difference between the length of hospital stay and the need for intensive care with the C-reactive protein value at 48 hours.
- Additionally, there was a statistically significant relationship between LAR and length of hospital stay, organ failure, and the need for intensive care.

## Introduction

Acute pancreatitis (AP) is a clinical condition characterized by the abnormal activation of pancreatic enzymes and the release of numerous inflammatory mediators, leading to sudden inflammation and destruction of the pancreas.^1^ Worldwide, gallbladder diseases and excessive alcohol consumption account for 80% of the etiology of AP.^2^ In Türkiye, the most common causes of AP are biliary, idiopathic, hypertriglyceridemia, and alcohol, in that order.^3^ Recently, in real-world data involving 2,144 patients, it was observed that 73% of AP patients experienced mild severity, 24% moderate severity, and 2.6% severe severity.^3^ Additionally, while the literature reports a mortality rate ranging from 6% to 10% for AP,^4^ a multicenter study conducted in Türkiye reported a lower rate of 1.6%.^3^ Severe AP can lead to serious complications and can be life-threatening, with a mortality rate of 10%-30%.^5^ Therefore, the early diagnosis of AP and the identification of factors that predict severe progression are crucial for planning patient follow-up and treatment approaches.

Many parameters have been investigated to predict the development of severe AP. These include pleural effusion, obesity, age, blood urea nitrogen, serum creatinine, hematocrit, C-reactive protein (CRP), procalcitonin, as well as scoring systems that incorporate multiple parameters such as the Ranson score, systemic inflammatory response syndrome (SIRS), bedside index of severity in AP (BISAP), and acute physiology and chronic health evaluation (APACHE-II) score.^6^ ^7^ Given its simplicity in calculation and comparability to the APACHE II score, the BISAP score is the recommended scoring system for routine clinical practice.^8^ The subsequently developed Sequential Organ Failure Assessment scoring system has also not met the need, as it is only suitable for intensive care patients and cannot be routinely used for all patients. In studies conducted, it has been stated that CRP, serum procalcitonin, and interleukin-6 (IL-6) tests can be used to predict severe AP.^9^

Neutrophils are the most abundant subtype of leukocytes in peripheral blood (40%-70%). In cases of systemic inflammation, their numbers increase above normal, while lymphocytes, another subtype of leukocytes, decrease as a physiological response. Neutrophil Lymphocyte Ratio (NLR) is an index determined by dividing these 2 hematological parameters and has been used as an indicator of inflammatory response in many studies. In cases of severe sepsis, bacteremia, inflammation, and stress, neutrophil counts increase while lymphocyte counts decrease. It has been shown in various studies that NLR provides significant results in predicting adverse outcomes compared to total white blood cell (leukocyte) count in many diseases such as coronary artery disease, esophageal cancer, colorectal cancer, and hepatocellular carcinoma.^10^^-^^13^ Therefore, NLR can be used to predict the prognosis of AP due to the inflammation that occurs in AP. In recent years, many studies have been conducted on the prognostic value of the NLR. Suppiah and colleagues have shown that an increased NLR effectively differentiates between mild and severe disease in patients with a diagnosis of AP.^14^ Another study indicated that NLR could serve as an early predictive factor for disease severity in patients diagnosed with AP, correlating with the Ranson score.^15^ In a retrospective study involving 490 cases of AP, 70 of which were severe, it was shown that a high NLR measured upon hospital admission and at 24, 48, and 72 hours post-admission was associated with the development of severe AP and organ failure.^16^

Lactate dehydrogenase (LDH) is an enzyme primarily involved in pyruvate-lactate conversion during glycolysis and in the oxidation of long-chain fatty acids in the liver. Serum LDH levels increase under conditions such as hemolysis, malignancy, infection, ischemia, tissue injury, necrosis, and hypoxia. Albumin, on the other hand, is a plasma protein synthesized by the liver and is the most abundant protein in the body. It is responsible for maintaining osmotic pressure, exerting antioxidant effects, and binding and transporting various substances into the blood. As a negative acute-phase reactant, albumin levels decrease under inflammatory conditions. Several studies have shown that the LDH/albumin ratio, calculated by dividing LDH by albumin, is an adverse prognostic marker for various diseases.^17^ ^18^

In this study, the aim was to demonstrate the relationship between the NLR, LAR, and the prognosis of AP more comprehensively by separately comparing NLR, LAR, and CRP with prognostic factors such as organ failure, intensive care requirement, length of hospital stay, and mortality, as well as the Harmless Acute Pancreatitis Score (HAPS), Ranson, and BISAP scoring systems. The aim was to determine the prognostic power of NLR and LAR in predicting severe AP and associated mortality, identifying which day’s measurement is more significant, and finding the cut-off value to consider it significant.

## Materials and Methods

**Figure 1.**
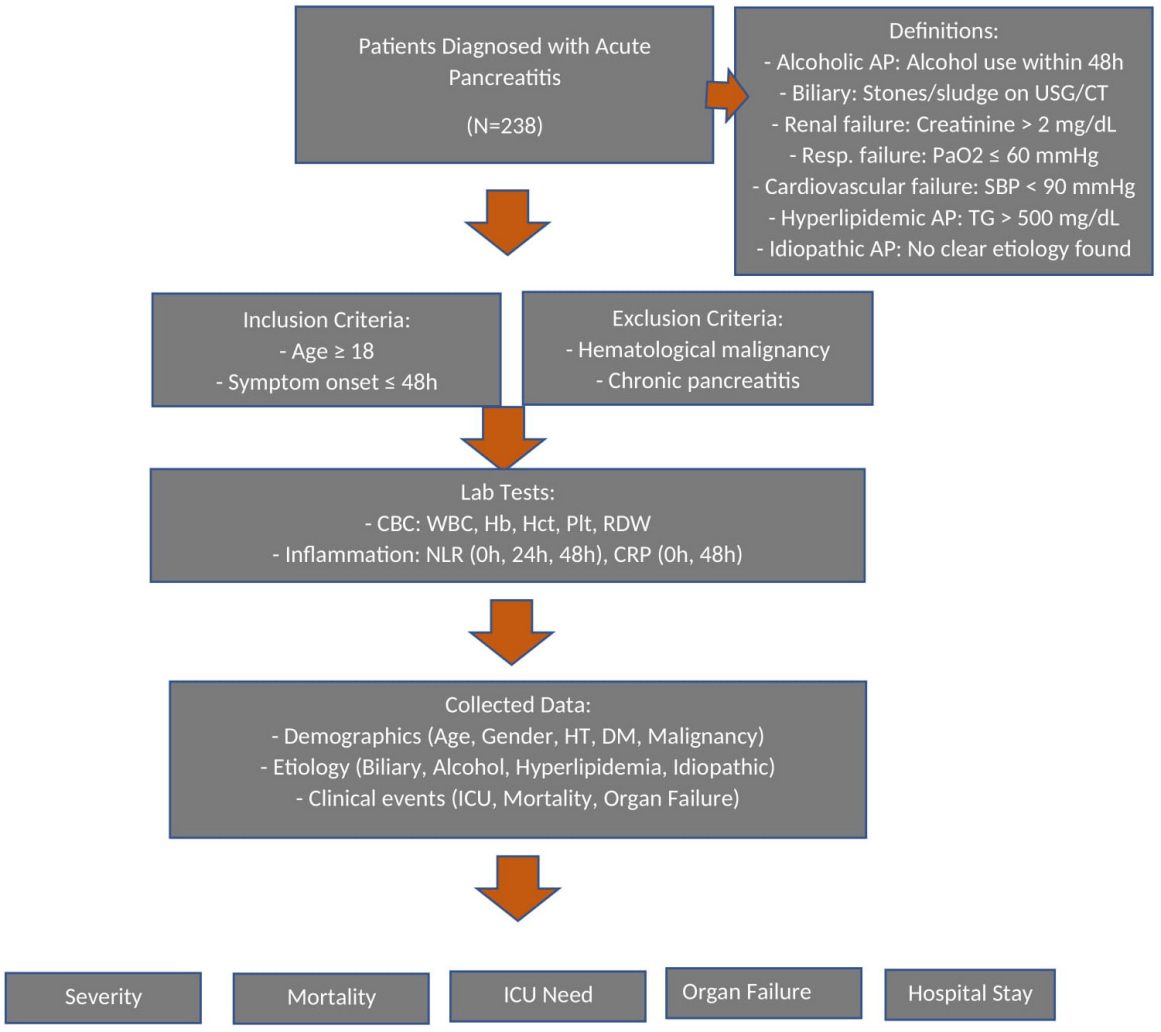
Flowchart of the study. Intensive care unit (ICU), Neutrophil (NLR), Lymphocyte Ratio (NLR).

This study is a single-center, retrospective study that examined the demographic, clinical, and laboratory data of 238 patients diagnosed with AP and treated at the Dokuz Eylül University Medical Faculty Hospital’s Gastroenterology Clinic between December 2021 and June 2023. The study included patients aged 18 and over who presented to the hospital within 48 hours of the onset of disease symptoms and for whom laboratory data were accessible during follow-up. Patients with hematological malignancies and a history of chronic pancreatitis were excluded from the study. Data collected from the patients included age, gender, history of malignancy, etiology of AP (biliary, alcohol, hyperlipidemia, other causes), presence of Diabetes, hypertension, need for antibiotics, organ failure, mortality, interventions (EUS or ERCP performed), length of hospital stay, need for intensive care, and laboratory tests including white blood cell (WBC) count, hemoglobin, hematocrit, platelets, RDW (Red Cell Distribution Width), NLR at the time of admission (0 hours), and at 24 and 48 hours, CRP values at 0 and 48 hours, plasma fasting glucose, BUN, creatinine, amylase, lipase, AST, ALT, GGT, ALP, LDH, albumin, and the LAR at 48 hours post-admission (Figure 1).

Additionally, the physical examination notes of patients at the time of emergency department admission were reviewed, and the Harmless Acute Pancreatitis Score (HAPS) was calculated within the first 30 minutes of admission to predict the severity of AP, along with the SIRS score in the first 24 hours, the BISAP score at 48 hours, and the Ranson score at the time of admission.

To classify the etiology of the included patients, for a diagnosis of alcoholic AP, it was required that the patient had consumed alcohol within the 48 hours prior to the onset of symptoms without any other cause to explain the etiology. A creatinine level exceeding 2 mg/dL within 48 hours was evaluated as renal failure, PaO2 ≤ 60 mmHg as respiratory failure, and a systolic blood pressure below 90 mmHg despite IV hydration as cardiovascular failure. A diagnosis of biliary AP was made based on the detection of biliary sludge or stones in ultrasound or computed tomography imaging. The need for antibiotics was determined by the presence of any infection in a system (pneumonia, cholangitis, urinary tract infection, significant microorganism growth in blood, urine, or other body fluid cultures), the presence of abscesses, infected pancreatic necrosis, or conditions indicating a high risk of infection meeting the SIRS criteria. Serum triglyceride levels above 500-1000 mg/dL are considered hyperlipidemia-related pancreatitis if no other cause can be identified. Cases of post-ERCP pancreatitis that develop following ERCP procedures, as well as cases of pancreatitis where the cause remains unidentified despite extensive investigations, are classified as idiopathic AP.

### Statistical Analysis

A database was created in IBM SPSS Statistics 21.0 for Windows (IBM SPSS Corp.; Armonk, NY, USA) for the descriptive analyses of the study, calculating the frequency, percentage, mean, SD, and median values of the data. Mortality and scoring systems were evaluated as dependent variables. The relationships between these dependent variables and all independent variables, which were count data, were analyzed using the Student’s t-test, and for non-normally distributed data, non-parametric tests were applied. Categorical variables such as age, gender, antibiotic requirement, need for procedures, malignancy, hypertension, diabetes mellitus, organ failure, intensive care unit (ICU) admission, SIRS, and length of hospital stay were analyzed using the Chi-square test. In the analysis, cutoff points for NLR at 0, 24, and 48 hours, the LDH/Albumin ratio, and the CRP value at 48 hours were calculated for sensitivity and specificity using ROC (Receiver Operating Characteristic Curve) curve analysis. Statistical significance was defined as *P* < .05.

Ethical approval for the study was obtained from the Dokuz Eylül University Non-Interventional Research Ethics Committee with decision number 2023/18-24 dated August 10, 2023. The study was conducted in accordance with the Declaration of Helsinki of the World Medical Association. Since the study was retrospective, informed consent could not be obtained from the patients.

## Results

A total of 238 patients diagnosed with AP between 2021 and 2023 were included in the study. Of the research group, 56.7% were women, with a mean age of 57.94 ± 16.83 years (range 20-93). Hypertension was detected in 45.0%, diabetes mellitus in 25.6%, and malignancy in 5.9% of the patients. Biliary stones were the etiology in 60.9% of the cases (Table 1). Regarding the AP severity scores, 10.9% of patients had high Ranson scores, 16.0% had high BISAP scores, and 14.7% had high HAPS scores. As shown in Table 2, mortality occurred in 3.8% of the research group, 6.7% required ICU admission, 34.9% had a hospital stay of more than 10 days, and 20.6% developed organ failure. Antibiotic use was necessary for 71.0% of the patients, while 66.8% did not require intervention during hospital admission. The mean total hospital stay was 12.46 ± 17.56 days (range 1-187).

The average NLR at admission (0-hour value) was 10.59 ± 11.30, while the NLR averages at 24 hours and 48 hours were 9.24 ± 11.49 and 7.96 ± 8.24, respectively. The average CRP values at 0 hours (day of admission) and at 48 hours were 82.27 ± 104.91 and 138.05 ± 113.21, respectively (Table 3).

No significant relationship was found between gender, etiology, need for intervention, antibiotic requirement, presence of diabetes mellitus, and AP severity according to the Ranson score. However, lower AP severity was detected in patients without hypertension or malignancy and those under 65 years of age. When analyzing the relationship between AP severity according to the BISAP score and demographic data, lower AP severity was also found in patients without hypertension or diabetes comorbidities, those without antibiotic requirements, and those under 65 years of age (Table 4).

Higher NLR values at 0, 24, and 48 hours and WBC levels were associated with higher AP severity according to both the Ranson and BISAP scoring systems, while high albumin levels were associated with lower AP severity only in the BISAP evaluation (Table 5). It was shown that NLR values at 24 hours and 48 hours could be the best parameters for predicting AP severity according to the Ranson score. Analyses using the Ranson, BISAP, and HAPS scoring systems demonstrated that both the NLR and CRP values at admission and at 24 and 48 hours play significant roles in predicting the severity of AP. According to the Ranson score, the sensitivity and specificity of NLR values measured at admission and at 24 hours in predicting AP severity were at satisfactory levels (NLR24 AUC (Area under curve) = 0.738, 72% sensitivity, 69% specificity). CRP48 also demonstrated similar performance (AUC = 0.671, 72% sensitivity, 63% specificity). According to the BISAP scoring system, the NLR achieved the highest sensitivity and specificity in predicting AP severity at 24 hours (NLR24 AUC = 0.823, 79% sensitivity, 67% specificity). The HAPS scoring system indicated that both NLR and CRP values were moderately successful in determining AP severity, particularly noting that NLR24 and CRP48 were significant prognostic indicators for predicting AP severity (CRP48 AUC = 0.746, 81% sensitivity, 66% specificity) (Table 6).

In this study, when evaluating the relationship between clinical and laboratory parameters and mortality, factors such as age, hypertension, and malignancy were found to have a significant impact on mortality. Specifically, a markedly higher mortality rate was observed in patients over 65 years of age and those with a history of malignancy *P* < .001). The presence of hypertension was also identified as an important factor affecting mortality (*P* = .044). Regarding laboratory results, the NLR values at admission (NLR0), at 24 hours (NLR24), and at 48 hours (NLR48) showed a strong relationship with mortality (*P* < .001). Similarly, WBC and albumin levels also demonstrated significant correlations with mortality (*P* < .001). Low albumin levels were found to be directly associated with increased mortality (Table 7).

When evaluating the effects of NLR and CRP values on prognostic parameters such as mortality, ICU admission, length of stay, and organ failure in AP patients, ROC analysis results indicated that NLR and CRP play a significant role in predicting these clinical outcomes.

- In terms of mortality prediction, particularly at 48 hours, NLR (AUC = 0.881, 85% sensitivity, 75% specificity) and CRP48 (AUC = 0.869, 85% sensitivity, 85% specificity) values showed high predictive power.
- For predicting ICU admission, NLR measured at 48 hours (AUC = 0.859, 86% sensitivity, 70% specificity) and CRP48 (AUC = 0.877, 86% sensitivity, 79% specificity) were identified as strong indicators of the need for ICU care.
- Regarding length of stay prediction, NLR48 and CRP48 values were found to be moderately successful in predicting prolonged hospital stays. The NLR48 (AUC = 0.786) and CRP48 (AUC = 0.799) values showed potential utility in identifying patients with extended lengths of stay.
- Regarding the prediction of organ failure, the NLR and CRP values showed low to moderate accuracy in predicting organ failure. The NLR value at 48 hours (AUC = 0.693) and CRP48 (AUC = 0.648) had limited prognostic value in determining the risk of organ failure (Table 8).

In this study, the LDH/Albumin ratio was also found to be an important marker for predicting mortality, organ failure, the need for intensive care, and length of hospital stay in patients with AP. It provided high accuracy in mortality prediction (AUC = 0.950, 88% sensitivity, 94% specificity). It was also an effective indicator for predicting the need for intensive care (AUC = 0.795). However, the predictive power of the LDH/Albumin ratio for organ failure and length of stay was found to be more limited (Table 9).

## Discussion

The overall mortality rate of AP is around 5%, while in severe cases, this rate can rise to 20%.^2^^,8^ Therefore, the use of scoring systems and prognostic markers to predict the severity of the disease is of great importance for treatment and prognosis.^19^ Although there are many scoring systems and laboratory parameters used to predict the prognosis of AP, none have fully met the need. Zahorec et al^20^ demonstrated that a marked increase in neutrophil counts accompanied by lymphocytopenia reflects immune response severity in critically ill patients, proposing the “neutrophil-lymphocyte stress factor” as a simple, routinely usable clinical indicator.^20^ In another large observational study, Azab et al^21^ evaluated 283 AP patients and found that NLR was significantly superior to total WBC count in predicting ICU admission and length of hospital stay. Specifically, patients with an NLR ≥7.6 had markedly worse outcomes, and NLR remained an independent predictor in multivariate models. In this study, according to the BISAP score, the best parameter for predicting severe AP was the NLR at 24 hours (cut-off: 8), while the NLR at 48 hours (cut-off: 9.7 and 6.3) provided the best results for predicting mortality and organ failure. The CRP value at 48 hours (cut-off: 213 and 126) was identified as the best parameter for predicting the need for intensive care and prolonged hospitalization. A significant relationship was found between malignancy, hypertension, low albumin, NLR, and CRP values with mortality. Additionally, the LDH/Albumin ratio has been demonstrated to be an important parameter that can be used in prognostic predictions.

In this study, biliary pancreatitis cases constituted 60.9% of all patients. According to the literature, the rate of biliary pancreatitis is 30% in the United States, Western Europe, and Asia, while in Türkiye, this rate can reach up to 70% due to the high prevalence of gallstones and relatively low alcohol consumption.^22^ ^23^ The rate of biliary etiology in this study is consistent with these data. In the study by Coşkun et al,^24^ the mortality rate was found to be 3.8%, which is the same as this study, where 9 of the 238 patients died.^24^

In the study by Lankisch et al,^25^ gender was not reported as an independent risk factor for mortality in AP. Similarly, in this study, there was no significant relationship found between gender and mortality, organ failure, intensive care needs, and length of hospital stay according to the Ranson and BISAP scores. A significant difference was found between advanced age (65 years and older) and AP severity. Moreover, a significant relationship was found between high BISAP scores and patients with hypertension, diabetes, or the need for antibiotics. The correlation between the severity of AP and advanced age, based on these 2 scoring criteria, may be due to the inclusion of age criteria in the scores or the possibility that the disease may present more severely in older patients. The relationship between advanced age and mortality in AP is also corroborated by the study of Koziel et al.^26^

In this study, in patients with high NLR and WBC counts, AP severity was found to be higher according to the Ranson and BISAP scores. In a retrospective study by Wang et al^27^ in 2017, which included 110 patients, a cut-off value of 10 was shown to distinguish between mild and severe AP. The advantage of this study over this one is that it includes a larger number of patients and demonstrates that the NLR values are correlated with AP severity at the initial presentation, as well as at 24 and 48 hours. Additionally, while Wang et al^27^ included only cases of hypertriglyceridemia-related AP, this study conducted a more comprehensive examination without distinguishing etiologically. The method difference arises from Wang et al’s^27^ classification of AP severity according to the Atlanta Classification, while in this study, AP severity was determined using the Ranson and BISAP scores and compared with NLR and CRP; due to the exclusion of radiological data, local complications could not be assessed. In this study, no statistically significant difference was found between RDW and AP severity according to Ranson and BISAP scores. Similarly, in the study by Yılmaz et al,^28^ no significant relationship was found between RDW and AP severity.

While the NLR parameters at admission, 24, and 48 hours, as well as the CRP value at 48 hours, showed a statistically significant relationship with mortality, no significant relationship was found between the CRP value at admission and mortality. A meta-analysis also indicated that CRP only provided predictive cut-off values for mortality in 2 studies.^29^ Factors that may explain this situation include CRP not being specific to pancreatic inflammation and its delayed peak plasma level.

In this study, 48-hour NLR (cut-off 9.7) was the best predictor of mortality, while 24-hour NLR (cut-off 9.4) and 48-hour CRP were most effective for assessing disease severity**. **In the study by Suppiah et al,^14^ similar findings were obtained, indicating that the NLR at 48 hours is an independent prognostic indicator in AP. However, in this study, the NLR at 24 hours was the most significant value for determining AP severity, while the most effective parameter for predicting mortality was the NLR at 48 hours. The difference between this study and that of Suppiah et al^14^ lies in the fact that, in addition to assessing AP severity, this study also explored the relationships between NLR and various prognostic parameters, such as mortality, ICU requirement, organ failure, and hospital stay duration. In a study by Gülen et al^30^ involving 322 patients, it was concluded that NLR was ineffective in determining mortality in the first 48 hours of the disease. This study also found that HAPS and RDW values had no predictive effect on mortality, with the only independent variable that could predict mortality developing within the first 48 hours being the Balthazar classification.

In this study, when assessing severe AP according to HAPS, the CRP cut-off value at 48 hours was determined to be 147.5 mg/L, providing 81% sensitivity and 66% specificity. In a retrospective study by Cardoso et al^31^ involving 379 patients, it was noted that the CRP value measured at 48 hours after hospital admission had good prognostic value regarding severe pancreatitis, pancreatic necrosis, and mortality, with a 48-hour CRP cut-off established at 170-190 mg/L. In another study by Basnayake et al,^32^ a CRP level greater than 150 mg/L measured 48 hours after the onset of AP was indicated to be a predictive marker for determining AP severity. The insignificant results of comparing the CRP value at admission with prognostic factors in this study may be due to the time taken for serum CRP levels to reach peak levels and the fact that CRP does not reflect severe inflammation at the onset of the disease. Additionally, the association of the CRP level at 48 hours with prognosis, along with the optimal cut-off value (147.5 mg/L) being determined similarly to other studies in the literature, suggests that high CRP levels several days after the onset of the disease could indicate a poor prognosis. In this study, when the cut-off values for CRP at 48 hours were taken as 213 mg/L and 126 mg/L, it was determined that CRP was a better parameter than NLR for predicting the need for intensive care and prolonged hospital stay. Azab et al^21^ determined the cut-off value for NLR at 4.8 in predicting hospital stay and intensive care needs in patients with AP.

For predicting organ failure, the best parameter was determined to be the NLR cut-off value of 6.3 at 48 hours, providing 70% sensitivity and 63% specificity. In a study involving 490 patients, the optimal cut-off values for NLR to predict AP severity and organ failure were determined to be 4.7 and 4.8, respectively.^16^ This study confirms that the NLR at 48 hours is an effective parameter for predicting organ failure.

The findings are consistent with previous studies supporting the utility of the NLR in predicting AP severity. A meta-analysis by Liu et al,^33^ which included 10 studies and over 1700 patients, reported that NLR has a moderately high diagnostic value in predicting severe AP, with an AUC of 0.82, sensitivity of 79%, and specificity of 71%—figures similar to those observed in the study. Furthermore, a large retrospective study by Sima et al,^34^ which evaluated over 700 patients, demonstrated that NLR measured at 48 hours (NLR48) had good predictive accuracy for mortality and severe disease, particularly in biliary and alcoholic pancreatitis, with an AUC of 0.81 for mortality prediction. These findings align with the results, where 48-hour NLR was found to be the most effective marker for predicting mortality and organ failure. However, their observation that NLR performed less accurately in hypertriglyceridemia-induced AP and in ICU-admitted patients suggests that the prognostic performance of NLR may vary with etiology and clinical setting—an important aspect for future research.

Lactate dehydrogenase, which is part of the Ranson criteria, is an important parameter for predicting poor prognosis in AP. It is emphasized that high LDH levels in AP can indicate severe AP due to both inflammation and local/systemic complications. In a retrospective study published by Xiang et al in 2020, the relationship between LDH and CRP levels and severe AP, multiple organ failure, and mortality was examined in 115 patients, determining a cut-off value of 235 U/L for LDH, indicating that LDH is a parameter reflecting poor prognosis and severe AP.^35^ Studies have shown that the LDH/Albumin Ratio, calculated by manually dividing the increased LDH by the decreased serum albumin levels in inflammatory conditions, can predict poor prognosis in gastrointestinal malignancies and Still’s disease.^17^ ^18^ In a study that prospectively evaluated 347 intensive care patients, the LAR was compared with the development of 30-day mortality and organ failure, and it was found to be higher in patients who developed organ failure and mortality.^36^ In this study, the cut-off value for the LAR was found to be significant for predicting mortality, with 88% sensitivity and 94% specificity when set at 139.5. This parameter is associated with severe inflammation and tissue damage in AP and can be used to predict severe pancreatitis.

Limitations of this study include being a single-center, relatively small sample size, and retrospective study, as well as the inability to demonstrate local complications due to the exclusion of radiological data. A strong aspect of this study is the simultaneous evaluation of multiple prognostic factors, such as mortality, intensive care needs, hospital stay duration, organ failure, and AP severity. Additionally, alongside NLR, the LAR was also examined, making the study the first to investigate the relationship between this ratio and mortality in patients with AP.

In conclusion, this study demonstrated that both the NLR and the LAR are reliable, cost-effective, and easily obtainable biomarkers that can assist in predicting the severity and prognosis of AP. Among the parameters evaluated, the 48-hour NLR was the strongest predictor of mortality and organ failure, while LAR also showed a significant association with adverse outcomes. By identifying effective cut-off values, this study provides additional insight into how these markers can be used in clinical practice. When combined with established scoring systems such as BISAP and Ranson, these biomarkers may improve early risk stratification and guide treatment decisions.

## Figures and Tables

**Table 1.** Demographic and Clinical Characteristics of the Study Group

		N	%
Age			
<65		152	63.9
≥65		86	36.1
Gender			
Female		135	56.7
Male		103	43.3
Etiology		
Biliary	145	60.9
	Non-biliary	93	39.1
Malignancy		
Yes	14	5.9
	No	224	94.1
HT		
Yes	107	45.0
	No	131	55.0
DM		
Yes	61	25.6
	No	177	74.4
Total		238	100.0

DM, diabetes mellitus; HT, hypertension.

**Table 2.** Distribution of Prognostic Factors in Acute Pancreatitis Patients

Prognosis	N	%
Mortality		
Yes	9	3.8
No	229	96.2
Hospitalization (days)		
≤ 10	155	65.1
>10	83	34.9
Intensive care requirement		
No	222	93.3
Yes	16	6.7
Organ failure		
Yes	49	20.6
No	189	79.4
Need for antibiotics		
No	69	29.0
Yes	169	71.0
Intervention		
EUS	34	14.3
ERCP	37	15.5
EUS+ERCP	8	3.4
No	159	66.8
Total	238	100.0

ERCP, endoscopic retrograde cholangiopancreatography; EUS, endoscopic ultrasonography.

**Table 3.** Averages of Neutrophil-Lymphocyte Ratio and C-Reactive Protein Values

	N	Minimum	Maximum	Mean	SD
NLR0	234	0.84	105.50	10.59	11.30
NLR24	228	1.00	128.00	9.24	11.49
NLR48	219	0.80	58.50	7.96	8.24
CRP0 (mg/L)	227	0.9	933.0	82.27	104.91
CRP48 (mg/L)	232	1.2	533.8	138.05	113.21

CRP, C-reactive protein; NLR, neutrophil-lymphocyte ratio.

**Table 4.** Relationship Between Acute Pancreatitis Severity According to Ranson and Bedside Index of Severity in Acute Pancreatitis Scores and Patient Characteristics

Variables	Severity of Acute Pancreatitis (Ranson)					Severity of Acute Pancreatitis (BISAP)				
		Low	High	Chi-square	*P*		Low	High	Chi-square	*P*
Age, years	<65	146 (96.1)	6 (3.9)	21.043	**<.001**	<65	144 (94.7)	8 (5.3)	35.916	**<.001**
	≥65	66 (76.7)	20 (23.3)			≥65	56 (65.1)	30 (34.9)		
Gender	Female	118 (87.4)	17 (12.6)	0.892	.345	Female	110 (81.5)	25 (18.5)	1.514	.218
	Male	94 (91.3)	9 (8.7)			Male	90 (87.4)	13 (12.6)		
Etiology	Biliary	127 (87.6)	18 (12.4)	0.846	.358	Biliary	117 (80.7)	28 (19.3)	3.093	.079
	Non-biliary	85 (91.4)	8 (8.6)			Non Biliary	83 (89.2)	10 (10.8)		
Procedure requirement	Yes	71 (89.9)	8 (10.1)	0.077	.781	Yes	70 (88.6)	9 (11.4)	1.844	.174
	No	141 (88.7)	18 (11.3)			No	130 81.8 ()	29 (18.2)		
Antibiotic requirement	Yes	148 (87.6)	21 (12.4)	1.351	.245	Yes	132 (78.1)	37 (21.9)	15.263	**<.001**
	No	64 (92.8)	5 (7.2)			No	68 (98.6)	1 (1.4)		
Malignancy	Yes	10 (71.4)	4 (28.6)	4.760	**.029**	Yes	11 (78.6)	3 (21.4)	0.331	.565
	No	202 (90.2)	22 (9.8)			No	189 (84.4)	35 (15.6)		
HT	Yes	86 (80.4)	21 (19.6)	15.127	**<.001**	Yes	77 (72.0)	30 (28.0)	21.111	**<.001**
	No	126 (96.2)	5 (3.8)			No	123 (93.9)	8 (6.1)		
DM	Yes	52 (85.2)	9 (14.8)	1.236	.266	Yes	44 (72.1)	7 (27.91)	8.661	**.003**
	No	160 (90.4)	17 (9.6)			No	156 (88.1)	21 (11.9)		

BISAP, bedside index of severity in acute pancreatitis; DM, diabetes mellitus; HT, hypertension.

**Table 5.** The Relationship Between the Severity of Acute Pancreatitis According to Ranson and Bedside Index of Severity in Acute Pancreatitis Scores and the Laboratory Values of the Patients

Variables	Severity of Acute Pancreatitis (Ranson)			Severity of Acute Pancreatitis (BISAP)		
	Low	High	*P*	Low	High	*P*
NLR0	9.44 ± 8.68	16.14 ± 12.93	**.001**	8.71 ± 8.12	17.73 ± 12.01	**<.001**
NLR24	7.91 ± 7.784	15.27 ± 10.11	**<.001**	7.37 ± 7.52	15.90 ± 9.05	**<.001**
NLR48	7.16 ± 7.53	14.43 ± 10.81	**<.001**	6.33 ± 5.94	16.22 ± 12.47	**<.001**
WBC	9098.07 ± 6626.02	14805.63 ± 8361.56	**.002**	8930.57 ± 6394.04	13884.80 ± 8771.11	**<.001**
Albumin	3.75 ± 0.56	3.67 ± 0.57	.473	3.79 ± 0.53	3.53 ± 0.68	**.010**
RDW	14.55 ± 2.01	15.10 ± 3.11	.226	14.52 ± 2.15	15.08 ± 2.16	.147

BISAP, bedside index of severity in acute pancreatitis; NLR0, neutrophil-lymphocyte ratio at the time of admission; NLR24, neutrophil-lymphocyte ratio measured at 24 hours; NLR48, neutrophil-lymphocyte ratio measured at 48 hours; RDW, red cell distribution width; WBC, white blood cell count.

**Table 6.** Calculation of Sensitivity and Specificity of Neutrophil-Lymphocyte Ratio and C-Reactive Protein Values According to Scoring Systems for the Severity of Acute Pancreatitis

	Cut-off	AUC (95% GA)	P	Sensitivity (%)	Specificity (%)
RANSON					
NLR0	8.6	0.683 (0.575-0.790)	.005	72	61
NLR24	9.4	0.738 (0.626-0.850)	<.001	72	69
NLR48	5.7	0.720 (0.599-0.840)	.001	81	56
CRP48	148.4	0.671 (0.562-0.779)	.009	72	63
**BISAP**					
NLR0	10.0	0.793 (0.705-0.880)	<.001	82	74
NLR24	8.0	0.823 (0.760-0.886)	<.001	79	67
NLR48	7.5	0.819 (0.749-0.888)	<.001	73	73
CRP48	160.0	0.767 (0.696-0.838)	<.001	73	69
**HAPS**					
NLR0	6.7	0.673 (0.587-0.759)	.002	81	56
NLR24	5.5	0.689 (0.595-0.784)	.001	81	50
NLR48	6.3	0.708 (0.615-0.801)	.000	75	62
CRP48	147.5	0.746 (0.650-0.842)	.000	81	66

BISAP, bedside index of severity in acute pancreatitis; CRP48, C-reactive protien value measured at 48 hours (mg); HAPS, Harmless Acute Pancreatitis Score; NLR0, neutrophil-lymphocyte ratio at the time of admission; NLR24, neutrophil-lymphocyte ratio measured at 24 hours; NLR48, neutrophil-lymphocyte ratio measured at 48 hours.

**Table 7.** Distribution of the Relationship Between Patient Characteristics and Mortality

Variables	Mortality			
		Yes	No	*P*
Age, years	<65	3 (2.0)	149 (98.0)	.052
	≥65	6 (7.0)	80 (93.0)	
Gender	Female	6 (4.4)	129 (95.6)	.539
	Male	3 (2.9)	100 (97.1)	
Procedure requirement	Yes	74 (93.7)	5 (6.3)	.476
	No	148 (93.1)	11 (6.9)	
ABT requirement	Yes	2 (2.5)	77 (97.5)	.648
	No	7 (4.4)	152 (95.6)	
Malignancy	Yes	3 (21.4)	11 (78.6)	**<.001**
	No	6 (2.7)	218 (97.3)	
HT	Yes	7 (6.5)	100 (93.5)	**.044**
	No	2 (1.5)	129 (98.5)	
DM	Yes	2 (3.3)	59 (96.7)	.811
	No	7 (4.0)	170 (96.0)	
Etiology	Biliary	5 (3.4)	140 (96.6)	.736
	Non-biliary	4 (4.3)	89 (95.7)	
Laboratory				
NLR0		22.04 ± 8.96	9.71 ± 9.180	**<.001**
NLR24		20.17 ± 11.15	8.30 ± 7.98	**<.001**
NLR48		20.19 ± 12.77	7.43 ± 7.61	**<.001**
WBC		17366.66 ± 7517.47	9421.12 ± 6872.05	**.001**
Albumin		2.99 ± 0.86	3.77 ± 0.53	**<.001**
RDW		15.10 ± 1.95	14.59 ± 2.17	.494

ABT, antibiotic; DM, diabetes mellitus, HT, hypertension; NLR0, neutrophil-lymphocyte ratio at the time of admission; NLR24, neutrophil-lymphocyte ratio measured at 24 hours; NLR48, neutrophil-lymphocyte ratio measured at 48 hours; RDW, red cell distribution width; WBC, white blood cell count.

**Table 8.** Calculation of Sensitivity and Specificity of Neutrophil-Lymphocyte Ratio and C-Reactive Protein Values for Predicting Mortality, Intensive Care Unit Admission, Length of Stay, and Organ Failure

	Cut-off	AUC (95% GA)	*P*	Sensitivity (%)	Specificity (%)
Mortality					
NLR0	11.5	0.852 (0.751-0.953)	.002	71	71
NLR24	8.00	0.869 (0.764-0.973)	.001	85	61
NLR48	9.7	0.881 (0.793-0.969)	.001	85	75
CRP48	255.5	0.869 (0.706-1.000)	.001	85	85
**ICU**					
NLR0	9.5	0.764 (0.670-0.858)	.001	80	65
NLR24	10.7	0.828 (0.760-0.897)	<.001	80	74
NLR48	7.5	0.859 (0.790-0.928)	<.001	86	70
CRP48	213.0	0.877 (0.791-0.964)	<.001	86	79
**Hospitalization**					
NLR0	6.0	0.630 (0.551-0.709)	.002	71	53
NLR24	5.5	0.748 (0.680-0.815)	<.001	82	60
NLR48	4.9	0.786 (0.722-0.850)	<.001	80	65
NLR48	5.9	0.786 (0.722-0.850)	<.001	75	70
CRP48	126	0.799 (0.735-0.863)	<.001	79	70
**Organ failure**					
NLR0	7,8	0.667 (0.574-0.759)	.001	70	60
NLR24	5,9	0.670 (0.575-0.764)	.001	70	54
NLR48	6,3	0.693 (0.598-0.788)	<.001	70	63
CRP48	108,6	0.648 (0.554-0.742)	.003	68	50

CRP48, CRP value measured at 48 hours (mg); ICU, intensive care unit; NLR0, neutrophil-lymphocyte ratio at the time of admission; NLR24, neutrophil-lymphocyte ratio measured at 24 hours; NLR48, neutrophil-lymphocyte ratio measured at 48 hours

.

**Table 9.** Calculation of Sensitivity and Specificity of the LDH-Albumin Ratio for Predicting Hospital Length of Stay, Intensive Care Unit Requirement, Organ Failure, and Mortality

LDH/Albumin	Cut-off	AUC (95% GA)	*P*	Sensitivity (%)	Specificity (%)
Mortality	139.5	0.950 (0.898-1.000)	<.001	88	94
Organ Failure	62.4	0.690 (0.597-0.782)	<.001	72	52
ICU Hospitalization	82.5	0.795 (0.670-0.920)	<.001	80	70
Hospitalization	52.0	0.632 (0.557-0.706)	.001	80	40

ICU, intensive care unit, LDH, lactate dehydrogenase.

## Data Availability

The data that support the findings of this study are available on request from the corresponding author.
